# Synergistic effect of mutagenesis and truncation to improve a polyesterase from *Clostridium botulinum* for polyester hydrolysis

**DOI:** 10.1038/s41598-018-21825-9

**Published:** 2018-02-27

**Authors:** Antonino Biundo, Johanna Reich, Doris Ribitsch, Georg M. Guebitz

**Affiliations:** 1 Austrian Centre for Industrial Biotechnology (ACIB), 3430 Tulln an der Donau, Austria; 2 Institute of Environmental Biotechnology, University of Natural Resources and Life Sciences (BOKU), 3430 Tulln an der Donau, Austria

## Abstract

The activity of the esterase (Cbotu_EstA) from *Clostridium botulinum* on the polyester poly(ethylene terephthalate) (PET) was improved by concomitant engineering of two different domains. On the one hand, the zinc-binding domain present in Cbotu_EstA was subjected to site-directed mutagenesis. On the other hand, a specific domain consisting of 71 amino acids at the N-terminus of the enzyme was deleted. Interestingly, a combination of substitution of residues present in the zinc-binding domain (e.g. S199A) synergistically increased the activity of the enzyme on PET seven fold when combined to the truncation of 71 amino acids at the N-terminus of the enzyme only. Overall, when compared to the native enzyme, the combination of truncation and substitutions in the zinc-binding domain lead to a 50-fold activity improvement. Moreover, analysis of the kinetic parameters of the Cbotu_EstA variants indicated a clear shift of activity from water soluble (i.e. *para*-nitrophenyl butyrate) to insoluble polymeric substrates. These results evidently show that the interaction with non-natural polymeric substrates provides targets for enzyme engineering.

## Introduction

Polyesters are present in our daily life and they find applications in many different areas from textiles to packaging^1–5^. Polyethylene terephthalate (PET) is one of the most widely used polyesters worldwide with an estimated annual global market of 50 Mtons^6^. Although PET was believed for a long time to be resistant to biodegradation due to the terephthalic acid building blocks, Yoshida *et al*. recently reported the ability of the bacterium *Ideonella sakaiensis* to degrade and assimilate PET^7^. Similarly, our group and others demonstrated hydrolysis of PET by enzymes belonging to the carboxylesterase family (EC 3.1.1). Recombinantly produced carboxylesterases allow targeted hydrolysis of the PET surface with the production of functional groups or the release of hydrolysis products to recover monomeric building blocks in recycling processes^8–16^. As in the case of *I. sakaiensis*, evolution can play a key role to increase the activity of enzymes on non-natural compounds^17^. In order to improve the activity of enzymes on non-natural substrates, a modification of specific amino acids and/or entire domains can be performed by site-directed mutagenesis (SDM). For instance, both approaches have been successfully used to improve the activity of an esterase from *Clostridium botulinum* (Cbotu_EstA), which was reported to be able to hydrolyze the aliphatic/aromatic copolyester polybutylene adipate-*co*-terephthalate (PBAT)^18^. This enzyme shares a similar overall structure with others members of the carboxylesterase family, the so-called α/β-hydrolase fold^19^. In general, the active sites of these enzymes, which contain the α/β-fold, are either buried in the enzymes or present on the surface. In the catalytic reaction, commonly a nucleophile (Ser, Asp, or Cys) residue, a His residue and an acidic (Asp or Glu) residue are involved. The nucleophile residue is situated at the tip of the so-called “nucleophile elbow”^20^. In the case of Cbotu_EstA, the active site is formed by Ser-182, His-426 and Asp-384. The 3D structure of Cbotu_EstA was reported to contain special features typical for I.5 lipase family which comprises another PBAT-hydrolyzing enzyme from the mesophilic species *Pelosinus fermentans* (PfL1)^21^. Members of the I.5 lipase family show industrially attractive features due to their ability to be active and stable at temperatures as high as 50–60 °C^22–24^. Like most lipases, they contain a lid structure which covers the active site of the enzyme^25^. In the presence of an interface, the lid domain is able to move and to perform the interfacial activation mechanism, increasing the activity of the enzyme by allowing the substrate to enter the active site^25–27^. Like commonly present in members of the I.5 lipase family, both enzymes, Cbotu_EstA and PfL1, contain a zinc-binding domain, previously found only in lipases from thermophilic organisms. A major difference between these two enzymes is the presence of an extra domain at the N-terminus of Cbotu_EstA, which is neither present in PfL1 nor in other members of the I.5 lipase family^28^. The building of the model lacking 71 amino acids from the N-terminus of Cbotu_EstA, showed the presence of a hydrophobic patch and higher dynamics of the lid for interfacial activation^28^. Interestingly, this truncated enzyme (del71Cbotu_EstA) was able to hydrolyze PET, in contrast to the wild-type enzyme Cbotu_EstA. On the other hand, mutations in the zinc-binding domain likewise led to higher activity on aromatic polyesters. Therefore, it was exciting to investigate whether the combination of the two modifications would synergistically improve the activity of this enzyme.

## Results and Discussion

### Engineering of the Zn-binding and N-terminal domains of Cbotu_EstA

**Figure 1:**
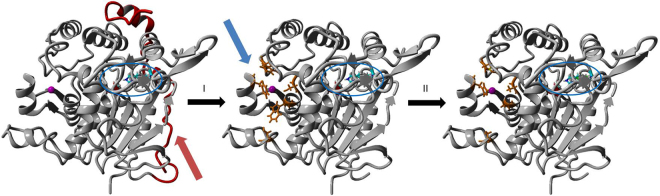
Concomitant engineering of two domains of Cbotu_EstA (PDB-Code 5AH1) to improve hydrolysis of PET. (**I**) The truncation of the N-terminal extra domain (red arrow) of 71 amino acids (marked in red) should lead to improved dynamics of the lid structure and the display of a hydrophobic patch for a better adsorption to hydrophobic substrates. (**II**) The substitution of residues (orange sticks) present in the zinc-binding domain (blue arrow) is expected to increase the activity of the enzyme on bulky water-insoluble substrates. The active site (Ser-182, His-426, Asp-384) is highlighted with a blue circle and the zinc ion in the zinc-binding domain in magenta. The representation was created using YASARA view (v. 14.7.17)^36^.

Based on the analysis of the 3D structure of Cbotu_EstA (Fig. 1), which was previously solved at a resolution of 1.2 Å, (PDB-Code 5AH1)^18^, we hypothesized that concomitant modification of the Zn-binding and N-terminal domain could improve the activity on the synthetic polyester PET. Individual modification in these regions has previously been shown to increase the activity of Cbotu_EstA on synthetic polyesters^28,29^. Substitution of amino acids present in the so-called entrance to the zinc ion cavity has an influence on the lid opening during interfacial activation in members of the I.5 lipase family such as the lipase from *Geobacillus stearothermophilus* (BTL2)^23^. On the other hand, Cbotu_EstA contains 71 amino acids at the N-terminus which covers the lid structure of the enzyme. Removal of this extra domain which is not present in other members of the I.5 lipase family would expose a hydrophobic patch which could improve the adsorption of the enzyme on hydrophobic surfaces and generally improve dynamics. Indeed, truncation of the N-terminal extra domain was shown to improve hydrolysis of polyester^28^. In this paper, these two approaches were combined to evaluate a potential synergistic effect on PET hydrolysis.

## Characterization of variants

**Figure 2:**
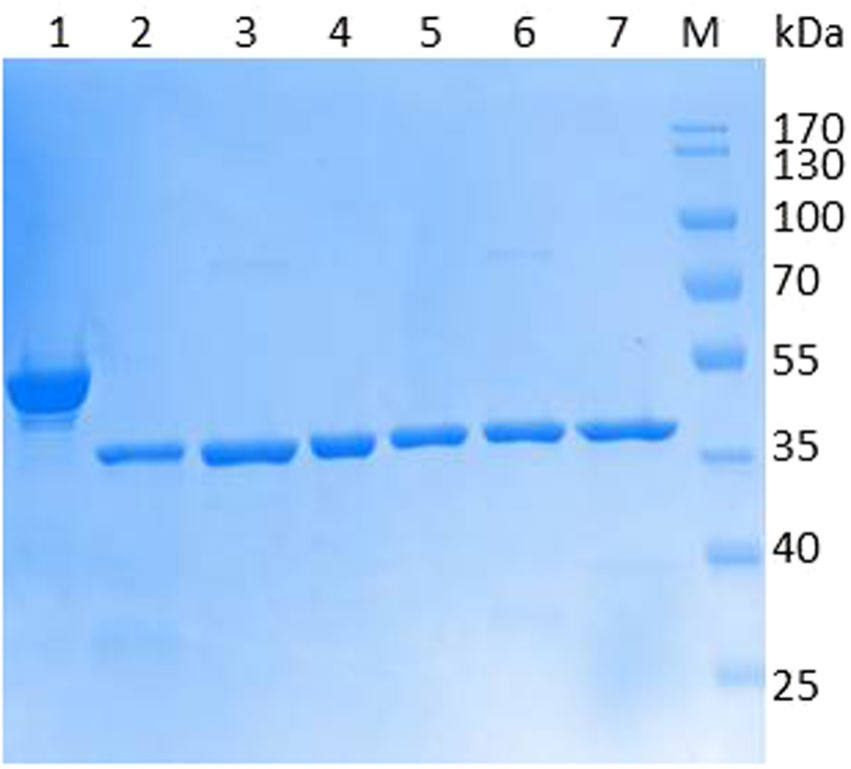
SDS-PAGE analysis (4–12%) of Cbotu_EstA wild-type and variants expressed in *E. coli* BL21-Gold(DE3) with 0.05 mM IPTG and purified by IMAC. *Lane 1*: Cbotu_EstA; *lane 2*: del71Cbotu_EstA; *lane 3*: del71Cbotu_EstA_S127A; *lane 4*: del71Cbotu_EstA_W129A; *lane 5*: del71Cbotu_EstA_F154Y; *lane 6*: del71Cbotu_EstA_S199A; *lane 7*: del71Cbotu_EstA_W274H; *lane M*: pre-stained protein molecular ladder Protein Marker IV (Peqlab, Germany). Image cropped and full-length gel found in Supplementary Information.

Plasmids of Cbotu_EstA variants without extra N-terminal domain and substitutions of residues present in the zinc-binding were transformed into *E. coli* BL21-Gold(DE3) and expressed at 20 °C with 0.05 mM IPTG to prevent the formation of inclusion of protein aggregation and precipitation^30,31^. SDS-PAGE analysis indicated a purity higher than 90% for all variants of Cbotu_EstA (Fig. 2).

**Figure 3:**
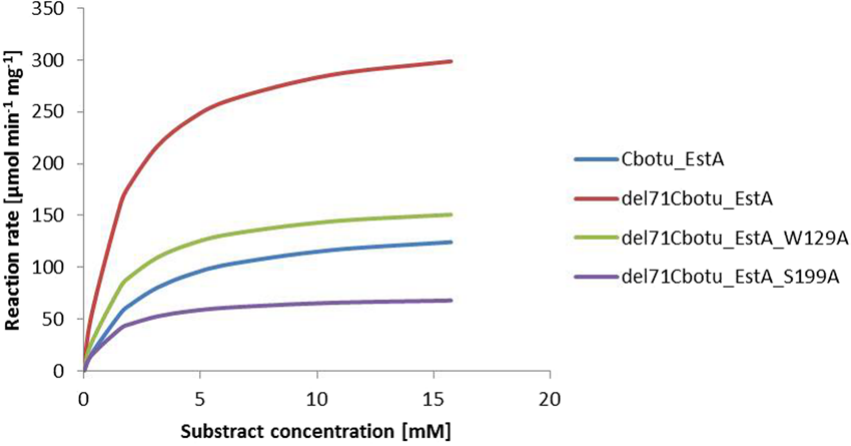
Michaelis-Menten plot of Cbotu_EstA, del71Cbotu_EstA, del71Cbotu_EstA_W129A, and del71Cbotu_EstA_S199A with *para*-nitrophenyl butyrate (*p*NPB) in a concentration range 0.3–15 mM. Michaelis-Menten plot of all the variants can be found in Supplementary Information (Fig. S1).

Kinetic parameters of the different variants were calculated (Figs 3 and S1). *K*_M_ values of the Cbotu_EstA variants were in the range between 0.60 and 2.48 mM (Table 1). The del71Cbotu_EstA variants showed similar values, while the variants containing the N-terminal domain showed the highest affinity towards *p-*NPB.

**Table 1:** Kinetic parameters of Cbotu_EstA wild-type and variants on the soluble substrate *p*-NPB.

	*K*_M_	*V*_max_	*k*_cat_	*k*_cat_/*K*_M_	Fold Change	References
	[mM]	[µmol min^−1^ mg^−1^]	[s^−1^]	[s^−1^ mM^−1^]		
Cbotu_EstA	2.5 ± 0.4	143.9 ± 6.9	123.3	49.7	—	This work
Cbotu_EstA_S127A	0.8 ± 0.1	23.9 ± 1.4	20.1	23.6	—	^29^
Cbotu_EstA_W129A	0.6 ± 0.1	12.5 ± 0.4	10.5	17.6	—	^29^
Cbotu_EstA_F154Y	1.1 ± 0.4	13.3 ± 0.8	11.2	10.3	—	^29^
Cbotu_EstA_S199A	1.2 ± 0.4	6.2 ± 0.5	5.2	4.5	—	^29^
Cbotu_EstA_W274H	0.8 ± 0.1	17.9 ± 0.5	15.1	19.3	—	^29^
del71Cbotu_EstA	1.6 ± 0.7	329.9 ± 38.8	238.6	145.5	2.9	This work
del71Cbotu_EstA_S127A	1.7 ± 0.7	242.6 ± 27.6	174.9	100.5	4.3	This work
del71Cbotu_EstA_W129A	1.6 ± 0.6	166.6 ± 17.0	120.5	73.5	4.2	This work
del71Cbotu_EstA_F154Y	1.2 ± 0.8	357.4 ± 53.1	258.5	210.1	20.4	This work
del71Cbotu_EstA_S199A	1.2 ± 0.5	73.4 ± 8.0	53.1	43.1	9.6	This work
del71Cbotu_EstA_W274H	1.0 ± 0.6	36.5 ± 4.4	26.4	25.2	1.3	This work

The ratio between the catalytic efficiency of truncated and 71 amino acids N-terminal domain-containing variants is shown as Fold Change.

Analysis of further kinetic parameters (*V*_max_, *k*_cat_ and *k*_cat_/*K*_M_) of del71Cbotu_EstA_S127A, del71Cbotu_EstA_W129A, del71Cbotu_EstA_S199A and del71Cbotu_EstA_W274H did not indicate increase in reaction rate, turnover number and kinetic efficiency towards the soluble substrate *p-*NPB, as contrarily shown for the other del71Cbotu_EstA variants.

**Figure 4:**
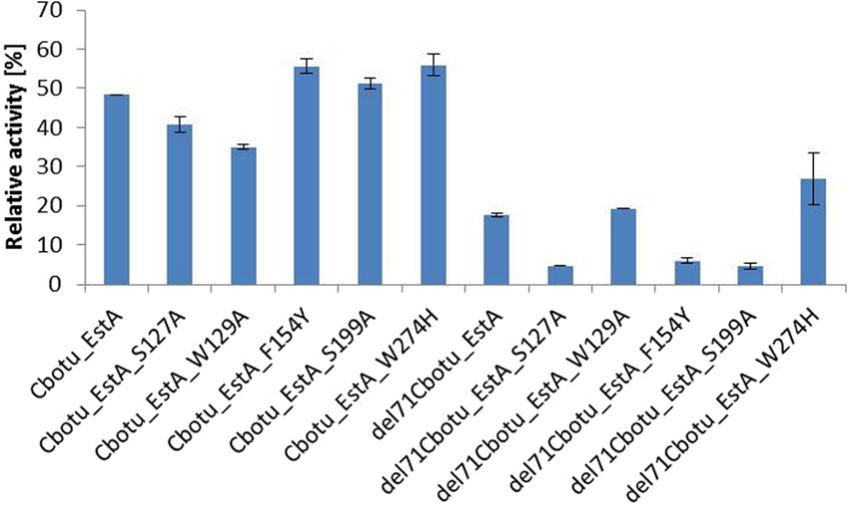
Thermal stability of Cbotu_EstA wild-type and variants determined with the soluble substrate *p*-NPB. The columns represent the residual activities of the purified enzymes after 24 h incubation at 50 °C.

The thermal stability of all del71Cbotu_EstA variants decreased when compared to the wild-type enzyme Cbotu_EstA and to the N-terminal domain zinc-binding domain variants. Those variants with substitution of Trp residue with either Ala or His residues (del71Cbotu_EstA_W129A and del71Cbotu_EstA_W274H) showed a higher thermal stability than other variants probably due to a lower hydrophobicity of the side-chain of the substituted amino acids improving solubility and the solvation by water molecules. After 48 h of incubation at 50 °C, only the two variants del71Cbotu_EstA_W129A and del71Cbotu_EstA_W274H showed residual activity of 7.2 and 16%, respectively, while the other variants completely lost their activity, including del71Cbotu_EstA (Fig. 4).

### Shift of Cbotu_EstA specificity from soluble to bulky substrates

**Figure 5:**
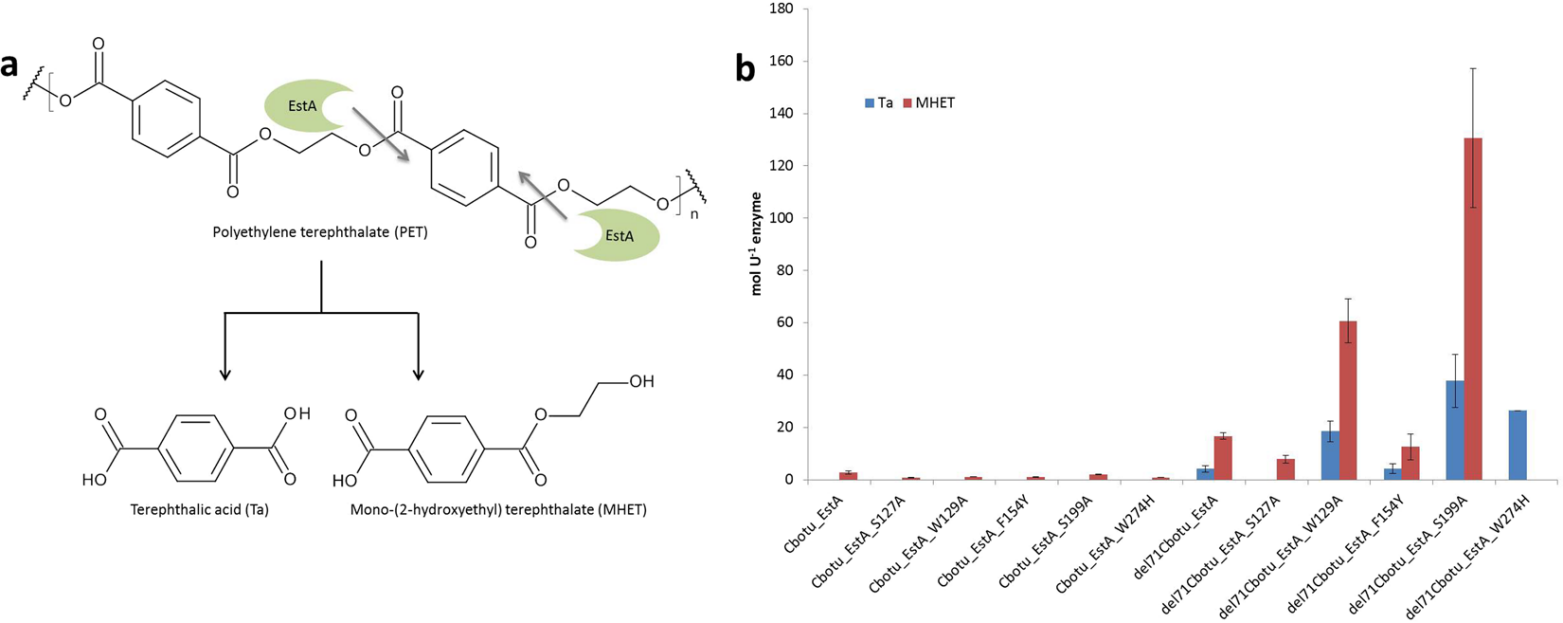
Enzymatic hydrolysis of PET by Cbotu_EstA wild-type and variants. (**a**) Possible PET hydrolysis pathway. The hydrolysis products Ta and MHET were detected by HPLC analysis, while the chromatogram was acquired at 241 nm. The grey arrow shows the possible cleavage site hydrolyzed by the Cbout_EstA variants. (**b**) PET hydrolysis of Cbotu_EstA wild-type and variants. The data are the mean value of three different measurements and the bars represent the standard deviation.

Elevated temperatures (50 °C) allow polymer chains present at the surface to be more motile, especially in amorphous regions, and hence improve enzymatic hydrolysis. Activity of the Cbotu_EstA variants on the bulky substrate PET at this temperature was quantified based on the release of terephthalic acid (Ta) and mono-(2-hydroxyethyl) terephthalate (MHET) (Fig. 5a). Cbotu_EstA showed no significant activity on PET most likely due to the low dynamics of the lid opening and the low surface hydrophobicity of the enzyme surface. In contrast, the truncated version of the enzyme, del71Cbotu_EstA, released some Ta and MHET from PET. Substitutions of the zinc-binding domain residues on the other hand did not improve PET hydrolysis when compared to the wild-type enzyme despite the fact that such mutations had previously been described to increase activity on other polyesters^29^ (Fig. 5b).

However, combination of both modification in variants del71Cbotu_EstA_W129A and del71Cbotu_EstA_S199A synergistically increased activity on PET by approximately four and seven times, respectively, when compared to del71Cbotu_EstA. Obviously, truncation of the extra domain at the N-terminus is necessary to allow the bulky PET to get in contact with the active site. Hence, only upon truncation, the mutations in the zinc-binding domain had an effect on PET hydrolysis. Both improved dynamics and exposure of a hydrophobic patch upon truncation of the extra domain at the N-terminus of the enzyme could improve interaction with the hydrophobic bulky substrate PET. Compared to the native enzyme, the combination of truncation and mutation in the zinc-binding domain increased the activity by a factor of 50. Interestingly, these two variants, del71Cbotu_EstA_W129A and del71Cbotu_EstA_S199A did not show significantly improved catalytic efficiency on the small substrate *p*-NPB while the activity on bulky PET was dramatically improved.

Furthermore, del71Cbotu_EstA_W274H showed a higher release of Ta as compared to both del71Cbotu_EstA and del71Cbotu_EstA_W129A, but a lack of MHET released (Fig. 5b). The successful tuning of substrate specificity from small water-soluble to large water insoluble substrates is clearly evident for del71Cbotu_EstA_W274H. This variant showed the lowest catalytic efficiency on *p*-NPB but a reasonable activity on PET when compared to the native enzyme.

## Conclusions

A novel strategy to tune the activity of the esterase Cbotu_EstA from small water soluble esters to bulky water insolubly polyesters was proposed. The combination of the substitution of Trp-129, Ser-199 and Trp-274 in the zinc-binding domain and the truncation of the N-terminal extra domain led to a synergistic improvement of the enzyme on PET when compared to variants carrying either of the individual modification. The discrepancy between the kinetics of the three variants, namely del71Cbotu_EstA_W129A, del71Cbotu_EstA_S199A and del71Cbotu_EstA_W274H, on the soluble substrate *p*-NPB and their activity on the bulky substrate PET clearly indicated a shift of enzyme specificity from the water-soluble substrate *p*-NPB to the water-insoluble polymeric substrate PET. Alhough thermal stability has been affected negatively especially in the case of del71Cbotu_EstA_S199A variant, and in general for the truncated variants, the combination of the substitution of Trp-129 and Ser-199 and Trp-274 improving dynamics together with the truncation of the N-terminal domain enhancing adsorption led to up to seven fold improved activity of the enzyme on PET, which shows highly active variants, compared to the wild-type enzyme Cbotu_EstA. Detailed analysis of the 3D structure of enzymes considering not only the active site architecture seems to be the key to improve activity on non-natural polymeric substrates such as the man-made polyester PET. Future studies could focus on the application of the concept presented here for a wider range of different polyesters as well as engineered enzymes for improved thermal stability. This would increase the potential of polyesterases both for surface functionalization and in recycling processes.

## Methods

### Chemicals, Reagents and Polymers

All chemicals and reagents used in this work were of analytical grade. Buffer components, *para*-nitrophenyl butyrate (*p*-NPB), bovine serum albumin (BSA) and HPLC-grade methanol were purchased from Sigma-Aldrich (USA). Films of polyethylene terephthalate (PET), with a thickness of 0.05 mm were purchased from Goodfellow, UK and used as polymeric substrate.

### General recombinant DNA techniques and site-directed mutagenesis

All DNA manipulations were performed by standard methods^32^. *Dpn*I restriction enzyme was purchased from New England Biolabs (USA). PCR was performed in a peqSTAR 96 Universal Gradient Thermocycler (Peqlab Biotechnologie GmbH, Austria) using *Pfu* DNA polymerase (Promega, Germany) with dNTPs purchased from MBI Fermentas (Germany). The plasmids were prepared using Promega mini- and midiprep kits (USA). DNA and protein sequences were analyzed by CLC Main Workbench, version 7.0.3 (Qiagen, Netherlands). Site-directed mutagenesis (SDM) of the mutants of Cbotu_EstA carrying mutations for the residues present on the zinc-binding domain was accomplished using the QuickChange multi site-directed mutagenesis kit (Stratagene) with plasmids previously prepared for each specific substitution^29^ and megaprimers for the deletion of the 71 amino acids at the N-terminus of each variant. The following megaprimers were designed so as to anneal on the vector (underlined) and on the desired gene sequence: 5′-TTG TTT AAC TTT AAG AAG GAG ATA TAC ATA GCA TTA TTG GTG GTA ACA ACT ATC CGA TTG-3′ (forward primer) and 5′-CAA TCG GAT AGT TGT TAC CAC CAA TAA TGC TAT GTA TAT CTC CTT CTT AAA GTT AAA CAA-3′ (reverse primer). The following PCR conditions were used: for the first step an initial denaturation was performed for 1 min at 95 °C, followed by 4 cycles for 50 s at 95 °C, with annealing for 30 s at 58 °C, and an extension for 9 min at 72 °C, aside from the final extension of 5 min. For the second step of PCR, 21 cycles instead of 4 were performed mixing the same amount of the two reactions together. In order to remove the template plasmid DNA, *Dpn*I treatment was carried out at 37 °C for 2 h, using 10 U of the restriction enzyme. The products were chemically transformed into *E. coli* XL-10 competent cells. The plasmids prepared from the positive clones were confirmed by commercial Sanger sequencing (LGC Genomics, Germany)^33^. The correct plasmids were chemically transformed into *E. coli* BL21-Gold(DE3) for further expression.

### Recombinant Expression, Protein Purification and Analysis

Freshly transformed *E. coli* BL21-Gold(DE3) cells were incubated and induced for protein expression as previously reported^29^. Purification was performed by IMAC as previously reported^21^. Fractions were pooled and concentrated by Vivaspin-20 column with a molecular weight cut-off (MWCO) of 10,000 Da following the manufacturer’s instructions (Sartorius AG, Germany). The buffer was exchanged with 0.1 M Tris-HCl pH 7 using PD-10 desalting columns (GE Healthcare, UK). The protein concentration of the concentrated enzyme solutions was measured using the Bio-Rad Protein Assay Kit (USA) following the Bradford protocol^34^ with BSA as protein standard. Sodium dodecylsulfate-polyacrylamide gel electrophoresis (SDS-PAGE) was performed following the manufacturer’s instructions, using a 4–15% gel. Pre-stained protein Marker IV was purchased from Peqlab (Germany) and used as a molecular mass marker. Proteins were revealed by Coomassie Brilliant Blue method.

### Standard Esterase Assay and Thermal Stability Measurements

The measurement of the activity of the wild-type enzyme and different variants was performed using *p*-NPB as substrate as previously described at 25 °C and pH 7^28^. Kinetic constants were determined in 100 mM potassium phosphate buffer pH 7 at 25 °C using substrate concentration in the range of 0.3 to 6.3 mM following the release of *p-*NP with absorbance at 405 nm (*ε*_*p-*NP_ = 8.31 M^−1^ cm^−1^) in a TECAN plate reader Infinite M200 PRO (Switzerland). Simple weighted non-linear regression was used to calculate parameters of the Michaelis-Menten equation using SigmaPlot software, version 12.5, with the Michaelis-Menten model (Systat Software Inc., Germany). Differences in thermal stability of different variants were determined at 50 °C, which was determined to be the optimal temperature of the wild-type enzyme Cbotu_EstA^18^ in 100 mM potassium phosphate buffer pH 7 for up to 24 h with 0.6 µM enzyme. Aliquots were withdrawn and tested with 100 µM *p*-NPB to measure the residual activity^35^. One unit of enzyme activity (U) was defined as the amount of enzyme releasing 1 µmol of *p-*NP per minute under the given experimental conditions. The specific activity was expressed as U mg^−1^ of protein.

### Hydrolysis of polyethylene terephthalate films

Pieces of 0.5 × 1 cm of PET films (thickness 0.05 mm) were cut and washed as previously described^28^. Hydrolysis experiments were performed using 2 µM enzyme in 1 mL potassium phosphate buffer pH 7 with the addition of one piece of PET film. The hydrolysis experiment was conducted for 72 h at 50 °C and 100 rpm. The reaction was stopped as previously described^8^. The hydrolysis products terephthalic acid (Ta) and mono-(2-hydroxyethyl) terephthalate (MHET) present in the supernatant were determined by high performance liquid chromatography (HPLC) as previously described^29^. A calibration curve of the chemicals was used to calculate the amount of hydrolysis products.

### Data availability statement

All data generated or analyzed during this study are included in this published article.

## Electronic supplementary material

Supplementary Information
