# Supplementary material for: Synergistic effect of mutagenesis and truncation to improve a polyesterase from Clostridium botulinum for polyester hydrolysis
Source: Sci Rep. 2018 Feb 27;8:3745. doi: 10.1038/s41598-018-21825-9 (PMC5829244; doi:10.1038/s41598-018-21825-9)
Supplement: Supplementary file 1 — Supplementary Information [file 41598_2018_21825_MOESM1_ESM.pdf]

Synergistic engineering approaches to improve a polyesterase from *Clostridium botulinum* for polyester hydrolysis

Antonino Biundo[1], Johanna Reich[2], Doris Ribitsch[1,2*], Georg M. Guebitz[1,2]

Full-length gel picture

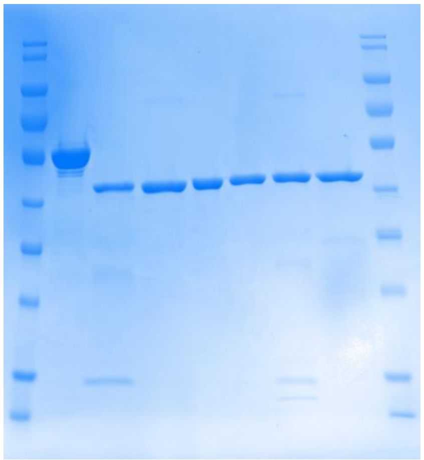

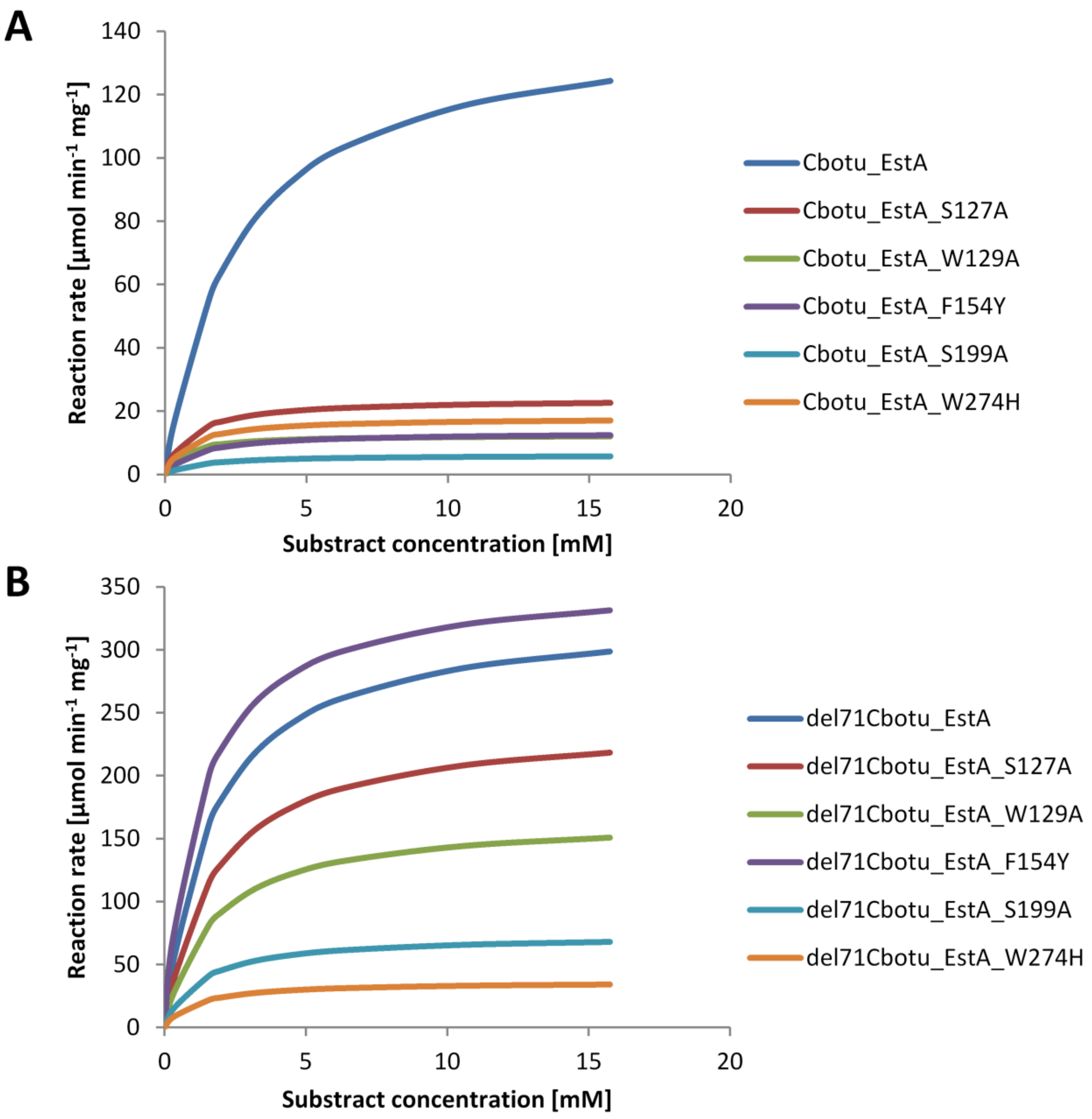

Figure S1. Michaelis-Menten plot of Cbotu_EstA wild-type and variants kinetics with the substrate *para*-nitrophenyl butyrate (*p*NPB) in a concentration range 0.3 - 15 mM. (**A**) Cbotu_EstA wild-type and variant from the zinc-binding domain. (**B**) del71Cbotu_EstA and variants from the zinc-binding domain.
